# Simulation of Orbital Fractures Using Experimental and Mathematical Approaches: A Pilot Study

**DOI:** 10.3390/jfb15060143

**Published:** 2024-05-26

**Authors:** Patrik Eiba, Karel Frydrysek, Behrad Zanganeh, Daniel Cepica, Pavel Marsalek, Petr Handlos, Juraj Timkovic, Jan Stembirek, Jakub Cienciala, Arnost Onderka, Michal Brezik, Ondrej Mizera

**Affiliations:** 1 Department of Applied Mechanics, Faculty of Mechanical Engineering, VSB—Technical University of Ostrava, 17. listopadu 2172/15, 70800 Ostrava, Czech Republic; patrik.eiba.st@vsb.cz (P.E.); karel.frydrysek@vsb.cz (K.F.); pavel.marsalek@vsb.cz (P.M.);; 2 Institute of Emergency Medicine, Faculty of Medicine, University of Ostrava, Syllabova 19, 70300 Ostrava, Czech Republic; 3 Faculty of Mechanical Engineering, Persian Gulf University, Khalij Fars St., Bushehr 7516913817, Iran; 4 Department of Forensic Medicine, Faculty of Medicine, Ostrava University, Syllabova 19, 70300 Ostrava, Czech Republic; 5 Department of Forensic Medicine, University Hospital Ostrava, 17. listopadu 1790/5, 70852 Ostrava, Czech Republic; 6 Clinic of Ophthalmology, University Hospital Ostrava, 17. listopadu 1790/5, 70852 Ostrava, Czech Republic; michal.brezik@fno.cz; 7 Department of Craniofacial Surgery, Faculty of Medicine, University of Ostrava, 70300 Ostrava, Czech Republic; 8 Clinic of Oral Maxillofacial Surgery, University Hospital Ostrava, 17. listopadu 1790/5, 70852 Ostrava, Czech Republic; 9 Laboratory of Molecular Morphogenesis, Institute of Animal Physiology and Genetics CAS, Veveri 97, 60200 Brno, Czech Republic; 10 Department of Machining, Assembly and Engineering Metrology, Faculty of Mechanical Engineering, VSB—Technical University of Ostrava, 17. listopadu 2172/15, 70800 Ostrava, Czech Republic

**Keywords:** biomechanics, traumatology, forensic science, splanchnocranium, experiment, modal analysis, fracture, orbital, static loading, dynamic loading

## Abstract

This contribution gives basic information about the mechanical behavior of the facial part of the human skull cranium, i.e., the splanchnocranium, associated with external loads and injuries caused mainly by brachial violence. The main areas suffering from such violence include the orbit, frontal, and zygomatic bones. In this paper, as a first approach, brachial violence was simulated via quasi-static compression laboratory tests, in which cadaveric skulls were subjected to a load in a testing machine, increasing till fractures occurred. The test skulls were also used for research into the dynamic behavior, in which experimental and numerical analyses were performed. A relatively high variability in forces inducing the fractures has been observed (143–1403 N). The results lay the basis for applications mainly in forensic science, surgery, and ophthalmology.

## 1. Introduction

In clinical medical practice, ocular trauma and periocular region injuries are caused by various trauma mechanisms that are accompanied by blunt force. It is, therefore, important to study the biomechanical features of the splanchnocranium. 

Orbital fractures are a relatively common type of injury accompanying the associated facial trauma from various causes. Most of these injuries are minor and do not require treatment, but some more serious, indirectly sight-threatening conditions require surgical intervention, which usually involves a multidisciplinary team of specialists including an ophthalmologist, a maxillofacial surgeon, and an otorhinolaryngologist [1,2,3,4]. Orbital fractures can be classified or subdivided based on many criteria, but, in general, three basic types are distinguished [5,6]. The first type includes fractures with disruption of the orbital rim (Le Fort II and III fractures, zygomaticomaxillary or nasomaxillary complex fractures, frontobasal fractures); the second type comprises retromarginal fractures, i.e., isolated orbital fractures with an intact orbital rim (blow-out fractures). The presence of a pneumatized sinus is a necessary condition for the development of this type of fracture [7,8]. The last basic type of orbital fracture, which is known as a blow-in fracture, is characterized by a dislocation of the fragments of the orbital floor inside the orbit. This type of fracture is, however, relatively rare [6].

The cause of the forces inducing the fracture may lie in active violence on the part of another person, such as a punch or a kick to the face or the orbit, as well as in dangerous injuries resulting from contact with solid protruding barriers, thrown objects, or fired projectiles; other accidents; wars; or sports. In addition to the superficial injuries of the skin cover, a complicated fracture of the facial skeleton (splanchnocranium) often occurs [9,10,11,12,13]. As a significant proportion of such injuries are caused by another person, the damage to health is often subject to subsequent criminal investigations and lawsuits. Elucidation of the mechanisms of the development of these injuries can, therefore, help in forensic investigations of such cases. Falls from a bike, scooter, or while inline skating are other relatively common causes of injuries to the splanchnocranium, and understanding the mechanisms of this injury could help in the development of protective devices that could minimize such injuries. Last but not least, data on the development of orbital fractures can be used when planning surgeries on the facial bones (selection of a suitable osteosynthetic material, location and direction of the osteosynthetic plates, etc.). 

Two generally accepted theories explain the formation of retromarginal fractures. The first considers them a result of a summation and transfer of forces to the orbital floor during a direct blow to the orbital rim. The other, known as hydraulic theory, explains the occurrence of retromarginal orbital fractures by the application of blunt force to the surface of the eyeball and its displacement in the anteroposterior axis, which is accompanied by a sudden rise in intraorbital pressure. The transfer of forces occurs on the principle of pressure transfer between the soft tissues and the bony shell and breaks the orbit at its weakest point, most often the orbital floor. Once the pressure in the orbit equalizes, the soft tissues return to their original position based on elasticity [7,8]. They may, however, be deformed and remain protruding into the maxillary sinus, which results in malposition and impaired ocular motility [5,6,9,11]. This theory is the most widely accepted mechanism of the formation of retromarginal orbital floor fractures. However, it does not reliably explain all cases of retromarginal fractures of the orbital floor. 

Clinically, the patient may also have a hematoma of varying size around the orbit; there may be no significant pain. These minimal clinical manifestations can sometimes lead to a failure to recognize this type of fracture, and can, therefore, result in the development of impaired ocular motility and, possibly, binocular diplopia within several months [10,14]. 

Historically, the hydraulic theory of retromarginal fracture formation has been more widely accepted, but it does not explain all cases of blow-out orbital fractures. It is apparently applicable only in situations where the apex of the eyeball is above the level of the imaginary junction of the superior and inferior orbital rims and/or in cases where the source of the applied violence is smaller than the orbital entrance. Therefore, a better understanding of the mechanism of retromarginal orbital fractures may help broaden the spectrum of indications for patient examination using computed tomography (CT), which is, together with a complex orthoptic examination, crucial for their diagnosis [1].

The presented work aims to experimentally elucidate the mechanism of retromarginal fractures of the orbital floor in a non-hydraulic environment (i.e., purely of the bony part of the head) and to describe the physical properties of the involved forces in more detail. This research included basic static and dynamic experiments, similar to previous studies by our team [15]. The static strength test of the zygomatic bone and modal analysis were experimentally performed on human cadaveric skulls. The numerical modal analysis employing the finite element method was calculated and compared with results from the experiment, and a prototype fixture (holder) was also developed for testing. The results of our study should provide initial data on the mechanics of orbital fractures purely from the perspective of the bony part. These results could serve as a basis for the future continuation of our experiments and research in the fields of biomechanics and forensics of these injuries. Such research could lead to improving the indication criteria for surgery, prediction of potential postoperative complications, and assessment of the forensic features of these complicated traumas.

## 2. Materials and Methods

### 2.1. Cadaveric Skulls

For our initial experimental research of biomechanical behaviors of the splanchnocranium, cadaveric nondamaged crania of adult men were used. Each cranium was used for two measurements (independently on the right and left orbit). These crania were from the depository of the Department of Forensic Medicine, University Hospital Ostrava (Ostrava, Czech Republic). All skulls had been osteologically processed in the past according to international standards [10]. The skulls were immersed in a 5% hydrogen peroxide solution, in which they were placed in a thermostat for 4 h and subsequently cleaned. If there was a risk of increased skeletal fragility in deceased persons over 60 years of age, the Hudec method was used, whereby maceration of the bone was carried out in one liter of distilled water in the presence of 80 g of lime chloride and 25 g of hydrochloric acid [15,16]. In three cases where the skulls were subsequently used for modal analysis, the cranial cavity was not opened. In the other three cases, the skull was opened. The crania were handled in accordance with ethical standards and scientific approaches [17]. Anthropometry parameters of skulls affect their mechanical properties. The type of measurement we have used for our tests is called euryon-to-euryon distance, which is equal to the maximum skull width.

### 2.2. Compression Test of the Cadaveric Skulls

The compression tests were performed on a Testometric M500-50CT testing machine (Testometric, Rochdale, UK).

**Figure 1:**
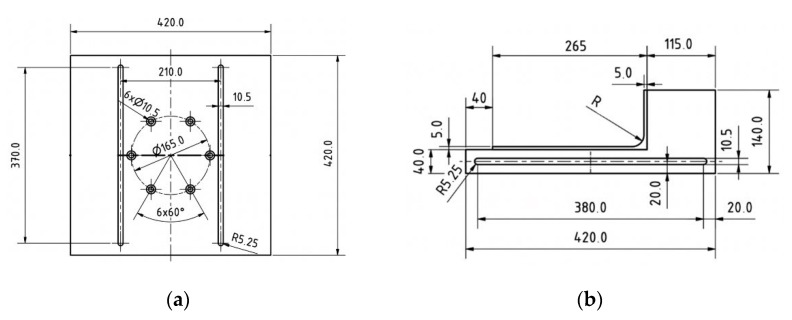
The dimensions of the adjustable holder (**a**) bottom plate and (**b**) side plate.

**Figure 2:**
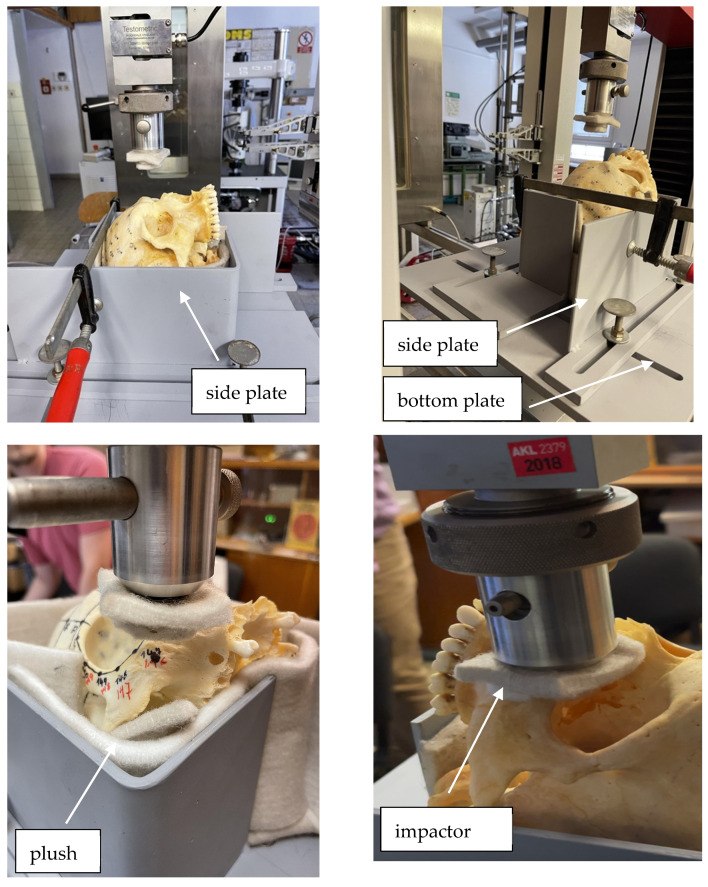
The adjustable holder of cadaveric skulls.

A special adjustable holder was designed and created for fixing the skulls in the desired position; see Figure 1 and Figure 2. The cadaveric skulls were fixed into the fixture, which was lined with a plush pad to partially simulate the soft tissues of the human body.

The fixture also included a metal impactor, which had been fitted with a plush pad at the point of contact with the skull; see Figure 2. The impactor acted as a surrogate for the human hand used in brachial violence. The quasi-static loading velocity was 10 mm/min, and the external load was increased until the splanchnocranial bones fractured. Acquired data were recorded for further processing.

### 2.3. Experimental Modal Analysis

**Figure 3:**
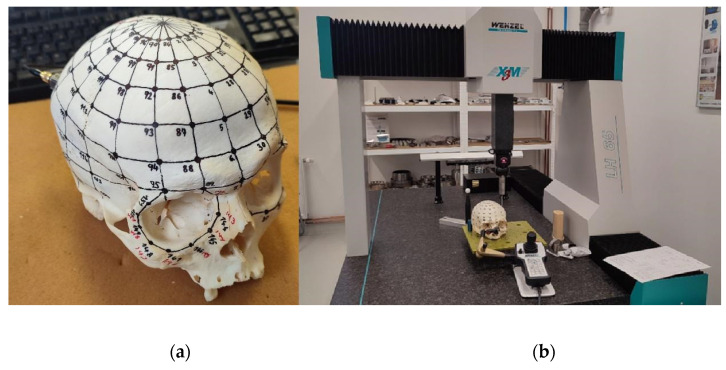
(**a**) The mesh of points representing the geometry of the skull; (**b**) measurement of the points of the network using the CMM scanner.

Experimental modal analysis was performed to obtain more information about the properties of the human skull’s structure. This is an experimental method for obtaining what are known as modal parameters, which can be used to mathematically describe the dynamic behavior of the measured structure. Modal parameters, namely eigenfrequencies, eigenshapes, and modal damping, acquired by experimental modal analysis of the skull, allow us to fit a numerical model using, e.g., the finite element method. This process requires high-accuracy measurement of modal parameters, which can be problematic for nonlinear or anisotropic materials. A human skull without the mandible was provided for the modal test. Since the shape of the skull is geometrically complex, a simplified regular grid of 151 points was created on the outer part of the skull; see Figure 3a. Coordinates in the Cartesian coordinate system were obtained for each point using a 3D CMM LH 65 X3 measuring device (WENZEL, Germany) with a Renishaw PH10M rotated head and a SP25M-1 measuring probe; see Figure 3b.

The tested cadaveric skull was mounted on a fixture and each point on the grid intersections was measured in space (three values corresponding to the axis coordinates). The sequential measurement was performed, following the sequence of the numbers on the skull in a transverse vertical direction. For specific areas on the cadaveric skull (temporal bone or sphenoidal bone), the head was tilted by 30 degrees. The software Quartis (Version software R2022-2) [18,19,20] was used for the evaluation of the measured points.

**Figure 4:**
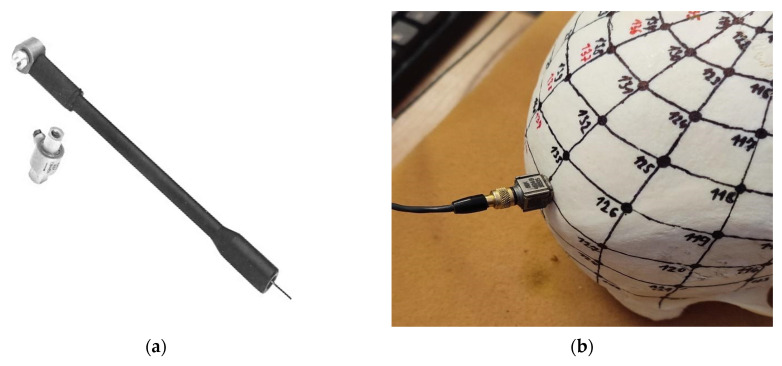
(**a**) B&K type 8203 impact hammer (**b**) and the B&K 4524B sensor/accelerometer in reference point.

Subsequently, the obtained point coordinates were imported into the modal analysis software BK Connect. To enable the visualization of custom shapes, the point mesh was filled with surfaces, thus creating a geometric model of the actual outer surface of the skull. For the measurements of modal parameters, a modal hammer excitation method with a B&K 8203 type force transducer (hammer) with a B&K 2647A type hub preamplifier was chosen; see Figure 4a. The excitation was performed at each nodal point of the grid, perpendicular to the skull surface. The response was measured at a fixed reference point using a B&K 4524B triaxial accelerometer; see Figure 4b.

The B&K 4524B sensor was also chosen due to its weight of about 4 g so as not to affect the measurement by inertial effects. The reference point was chosen at the back of the skull outside the longitudinal plane. A specially formulated beeswax, supplied by B&K, was used to attach the accelerometer to the skull. The attachment of the sensor using this wax was verified in the measured frequency range. The choice of the fit of the measured structure is an important point in the preparation of the experiment. If the measured data are used for comparison with a model created by, for example, the finite element method, a “loose fit” is ideal. In the case of the modal skull testing, a soft foam pad was used. It is clear that the fit on the soft pad should not ideally be loose as a bond is created between the skull and the pad. However, in the case of a relatively rigid skull, this fact does not affect the resulting values of the deformation of natural frequencies or natural shapes. The B&K analyzer with a four-channel module type 3160-A-042 was used to measure and process the signals. Due to the frequency range of the response sensor used, the analysis was set to the 3200 Hz range with sufficient sampling of 6400 lines. The number of averages was set to 5, which means that each point of the measurement grid had to be built up by the modal hammer 5 times. It was necessary to observe the exact direction and location of the structure excitation during the measurement. To control the individual measurements, the value of the coherence function, which ideally takes the value of 1 over the entire frequency range, was monitored for each average. The values of the coherence function dropped significantly at some of the measured points, especially in the regions of the cranial sutures and cheekbones, where bone homogeneity is most disturbed. However, the results were usable.

### 2.4. Numerical Model of Modal Analysis

To solve the undamped eigenvalue problem in mechanics or biomechanics, the following equation can be used:

$$\left(K-{\Omega_{i}}^{2}M\right)\phi_{i}=0,$$

where K represents the stiffness matrix, M represents the consistent mass matrix, ${\Omega_{i}}^{2}$ represents the square of the natural frequency, $\phi_{i}$ represents the mode shape, eigenfrequency is related to the eigenvector; the solver LS-DYNA R13, Share Memory Parallel (SMP) with double precision (Ansys, Canonsburg, PA, USA) can be used. Based on the complexity of the task, the solver uses BCSLIB-EXT 4.1 Library (Boeing Co., Arlington, TX, USA) to search for the first three eigenfrequencies and eigenvectors from 1 Hz using the Block Shift and Invert Lanczos Algorithm. The details of the sparse eigenvalue solver in LS-DYNA are described by Hallquist in the theory manual [20].

**Figure 5:**
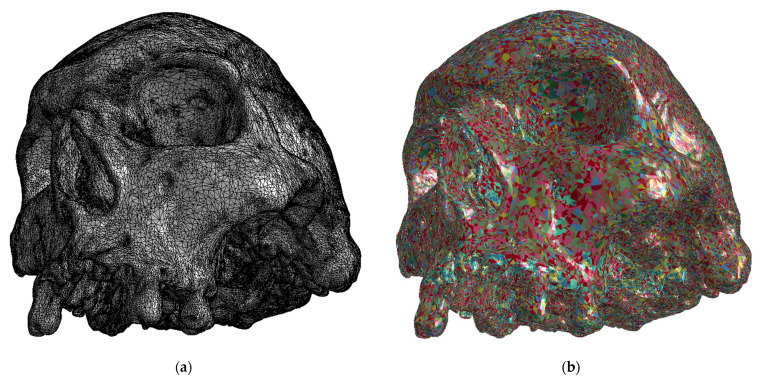
(**a**) Finite element mesh and (**b**) material inhomogeneity of the cadaveric skull.

Stiffness and mass matrices were constructed in the image processing software Materialise Mimics (Materialise NV, Leuven, Belgium), which created a finite element mesh using images obtained from a Computed Tomography (CT) scan; see Figure 5a. The mesh is formed by tetrahedral elements with quadratic displacement approximation. The finite element mesh statistic is presented in Table 1.

The cadaveric skull was divided into 100 regions, for which a linear elastic material model was assumed; see Figure 5b. Each region was described by Young’s modulus and density (calculated on the basis of the radiological results of the CT scan, the statistic values see Table 2). Poisson’s ratio was the same for all regions. Its value μ = 0.19 was taken from the paper published by McElhaney et al. [21]. A sensitivity analysis of the obtained material constants was performed. A sensitivity analysis was performed by calculating the numerical models, in addition to the measured values, also for 95% and 105% of the measured values of the Young’s modulus and density.

The proposed numerical model did not include the effect of damping. The effect of the initial stress state caused by the cadaveric cranium’s own weight was not implemented here. The task was solved on a standard machine (Intel i9-12900K workstation, 16 CPU, 128 GB RAM), with a calculation time of 345 min.

## 3. Results and Discussion

### 3.1. Static Compression Test Results for Cadaveric Crania

As mentioned in Section 2.1, six undamaged cadaveric crania of adult men were used. Each cranium was used twice, acquiring independent information on loading and breaking the right and left orbit. In total, therefore, twelve quasi-static compression experiments were performed on chosen specimens of human cadaveric crania. From each experiment, the dependence of external force on the displacement of the impactor was acquired.

All tests ended with a fracture of the zygomatic bone close to the orbital process. During the test of each specimen, a minor fissure appeared at first. With a further increase of the load, a full fracture of the zygomatic bone and other parts of the splanchnocranium occurred. The fracture and its initiation were always associated with the typical sound effects of cracking. The initiation of the fracture was always the same, regardless of the chosen side of the human skull.

**Figure 6:**
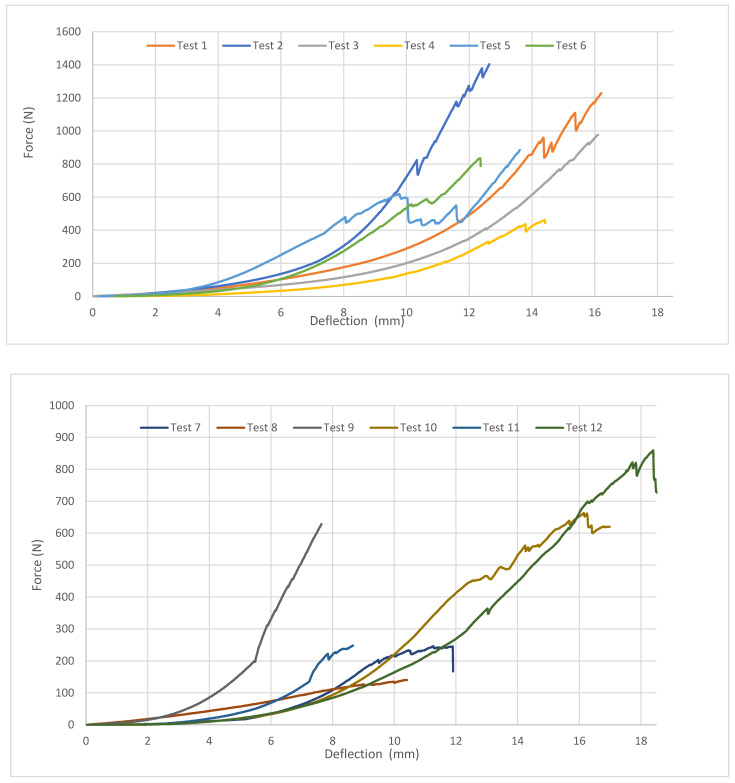
The compression test results of cadaveric skulls, measurements 1-12.

The summary of the results is clearly displayed in Table 3 and in Figure 6. The measured data showed significant variability in the external maximum loading force required for the development of the initial fissure, ranging from 136 to 953 N. Forces necessary for the development of the full fracture of the orbit ranged from 143.6 to 1403.6 N. This variability seems to be associated with age.

**Figure 7:**
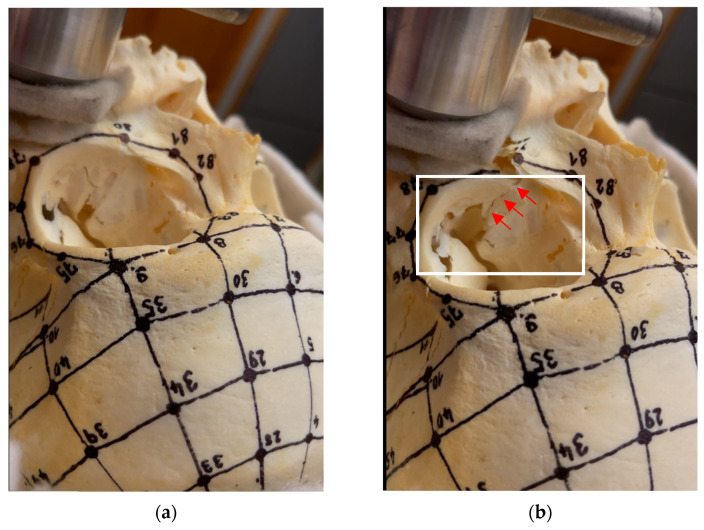
Cadaveric skull—(**a**) beginning of the compression test (**b**) final condition after the test with fracture (red arrows).

Figure 7 represents the results from compression tests, showing two phases of the experiment. The first (a) is the beginning of the test without any damage and the other (b) shows the final fracture. In all tests, the first signs of the fracture appeared on the zygomatic bone and continued to the final fracture. The differences between phases (a) and (b) are highlighted.

### 3.2. Dynamic Results of the Experimental Modal Analysis

**Figure 8:**
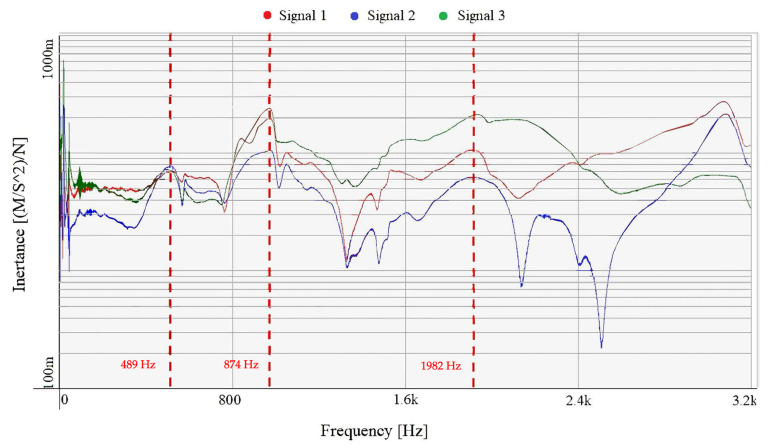
Frequency response function of the cadaveric cranium.

Two natural frequencies (489 Hz and 874 Hz) are relatively well evident from the results of the experimental model analysis of the skull, see Figure 8. A third frequency (1982 Hz) was less obvious, but, judging by the 180° change in the phase diagram, we assume that it is also a natural frequency. All three natural frequencies are highlighted in red in the graph.

It should be noted that due to the complex structure of the skull, the measurement is burdened with possible error. For this reason, only natural frequencies are presented here, which are—unlike, for example, modal damping values—not as significantly affected by measurement error. In order to evaluate the damping with sufficient accuracy, it would be advisable to perform measurements with a larger number of diameters and preferably on a larger number of samples. Using a frequency exciter instead of an impact hammer could be a possible alternative way of measurement that could provide better results, as that method allows full control of the input signal. For the validation of a possible numerical finite element method model of the skull, the presented results are, however, sufficient.

**Figure 9:**
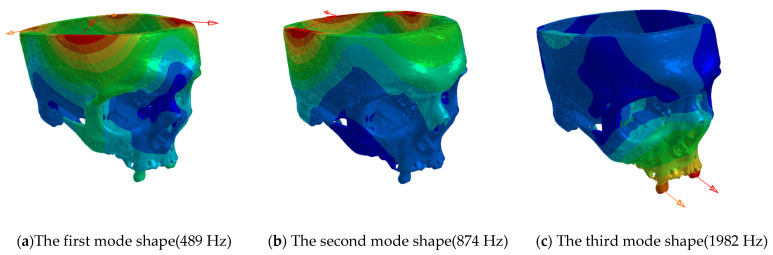
The first three mode shapes, the red arrows showing the movement of the cadaveric skull.

It needs to be mentioned that the properties are quite different in the case of a living human since the natural frequency values are influenced not only by stiffness but also by mass, and, once soft tissues are taken into account, the modal parameters will be different. Still, it should be noted that our study did not aim to fully elucidate the mechanics of orbital fractures but rather to provide initial data on the mechanics of the bony part. We expect to build on these data in the future by adding soft tissues for studies on the biomechanics and forensics of these injuries. Such research could lead to improving the indication criteria for surgery, prediction of potential postoperative complications, and assessment of the forensic features of these complicated traumas (Figure 9).

### 3.3. Future Research

This paper reports the initial results of our research. Human bones are a strongly anisotropic material whose biomechanical properties are dependent on the material distribution, age, sex, and method of loading. This variability is also evident in our statistical data. The compression tests were carried out on male cadaveric skulls only. Future testing should also include female cadaveric skulls to allow comparison of the results. Cadaveric skulls of men aged 55–80 were the most represented in our data. The results showed us that the strength of the skulls of younger individuals was generally much higher. From these results, we can observe that age has a significant influence on the strength of the zygomatic bones. To prove this observation, however, more compression tests on younger cadaveric skulls are needed; it is also important to state that there is a high variability even among individuals of the same age. 

The compression tests, which simulated brachial violence or being hit with projectiles, were performed using a metal impactor focused on the zygomatic bone. However, the test conditions were simplified compared to the real-world situation and focused only on the bony part. The muscles, skin, body fluids (such as blood), and, particularly, the eye were, therefore, neglected in our experiments. Still, the information on the behaviors of the skull can be utilized and serve as a basis for future experiments in which we could use, for example, a small rubber ball inserted into the orbit. This simulated eye will, during the compression, generate pressure on surrounding structures and may affect the resulting forces or even cause fractures in other places. 

The speed of loading is very important and can significantly affect the damage to the skull. All our compression tests on cadaveric skulls were performed with quasi-static loading. At higher loading speeds, material properties change; for a better understanding of the behavior of cadaveric skulls with different loading speeds, more tests, such as high-velocity impact tests, should be performed. 

**Figure 10:**
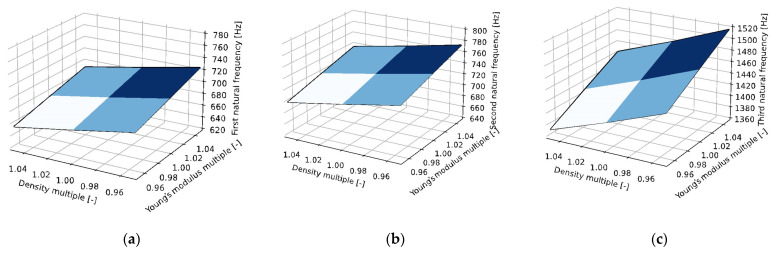
Surface expressing the effect of the eigenfrequency of a cadaveric skull on the change in density and modulus of elasticity ((**a**) the first eigenfrequency, (**b**) the second eigenfrequency, (**c**) the third eigenfrequency).

As for the experimental modal analysis, we can say it is very hard to measure the real response of a cadaveric skull due to its anisotropic behavior see the surface expression of the eigenfrequency results in Figure 10. However, we successfully determined the first three natural frequencies. The measured data were compared with the finite element model, showing relatively high differences see Table 4. In our future research, we plan to perform experimental modal analyses on a higher number of cadaveric skulls to acquire more robust results and compare those results with finite element models of all these skulls as well as with anthropometric data, which would further enhance our understanding of the biomechanical properties of the splanchnocranium.

The data we acquired in this study can be used for the application of numerical methods, further experiments, forensic science, application of dynamic loads, stochastic loads and modeling, or modeling of bones as a composite material, thus supporting interconnections with clinical implications beyond a single medical field or biomedical engineering. The results are also applicable in implantology in osteosynthesis of splanchnocranial fractures, facial surgery, and ophthalmology. 

## 4. Conclusions

In our study, we performed static loading tests as well as experimental modal and finite elements method analysis of human skulls, eliciting pressure on the zygomatic bone. Loading this point resulted, in all skulls, in the fracture in the zygomatic bone close to the orbital process. The forces necessary for the development of a fracture were highly variable even within the measurements in the same skull; our results also suggest that fragility is age-related. The comparison of the experimental and numerical modal analyses revealed relatively large differences, indicating the need for further investigations. Still, the obtained results can be used as a basis for further research that could eventually lead to designing implants for the treatment of complicated fractures in maxillofacial and ocular surgery or new therapeutic procedures; the findings could be also applicable in forensic science in determining and evaluating the degree of brachial violence or in improving and updating numerical and experimental approaches in biomechanics.

## Figures and Tables

**Table 1:** Statistics of the finite element mesh.

Number of Nodes	Number of Elements	Number of DOF
3,898,015	2,535,958	11,694,045

DOF—Degrees of Freedom.

**Table 2:** Statistics of the finite element mesh.

Young’s Modulus	Density	Poisson’s Ratio
Min. 123.686 MPa	Min. 20.812 kg/m^3^	0.19
Max. 24,552.3 MPa	Max. 4144.3 kg/m^3^	0.19
Mean 12,231.7 MPa	Mean 2064.4 kg/m^3^	0.19
Std. deviation 7166.7 MPa	Std. deviation 1209.6 kg/m^3^	0
Median 12,214.6 MPa	Median 2061.2 kg/m^3^	0.19

**Table 3:** Acquired loading forces; note that in all tests, the fracture developed in the zygomatic bone close to the orbital process.

Test No.	Initiation Force of Fracture [N]	Max. Loading Force [N]	Age	Sex
1	820	1229	20–30	Male
2	953	1403	20–30	Male
3	819	977	20–30	Male
4	404	461	20–30	Male
5	461	885	55–65	Male
6	389	834	55–65	Male
7	129	246	65–80	Male
8	136	143	65–80	Male
9	431	628	55–65	Male
10	455	650	55–65	Male
11	205	242	65–80	Male
12	794	860	65–80	Male
Median	819	1103	Individuals 20–30	
Maximum	953	1403		
Minimum	404	461		
Median	443	742	Individuals 55–65	
Maximum	461	885		
Minimum	389	628		
Median	170	244	Individuals 65–80	
Maximum	794	860		
Minimum	129	143		

**Table 4:** The results of sensitive analysis.

Natural Frequency	Young’s Modulus Multiple	Density 95%	Difference from Experiment	Density 100%	Difference from Experiment	Density 105%	Difference from Experiment
	95%	688.3 Hz	40.8%	670.9 Hz	37.2%	654.7 Hz	33.9%
Frequency 489 Hz	100%	706.2 Hz	44.4%	688.3 Hz	40.8%	671.7 Hz	37.4%
	105%	723.6 Hz	48.0%	705.3 Hz	44.2%	688.3 Hz	40.8%
	95%	734.3 Hz	−16.0%	715.7 Hz	−18.1%	698.5 Hz	−20.1%
Frequency 874 Hz	100%	753.4 Hz	−13.8%	734.3 Hz	−16.0%	716.6 Hz	−18.0%
	105%	772.0 Hz	−11.7%	752.4 Hz	−13.9%	734.3 Hz	−16.0%
	95%	1441.7	−27.3%	1405.2	−29.1%	1371.4	−30.8%
Frequency 1982 Hz	100%	1479.2	−25.4%	1441.7	−27.3%	1407.0	−29.0%
	105%	1515.7	−23.5%	1477.3	−25.5%	1441.7	−27.3%

## Data Availability

The raw data supporting the conclusions of this article will be made available by the authors on request.
